# Oxidative Stress in Psoriasis Vulgaris Patients: Analysis of Asymmetric Dimethylarginine, Malondialdehyde, and Glutathione Levels

**DOI:** 10.3390/medicina61060967

**Published:** 2025-05-23

**Authors:** Neşe Göçer Gürok, Selda Telo, Büşra Genç Ulucan, Savaş Öztürk

**Affiliations:** 1 Department of Dermatology, Fethi Sekin City Hospital, University of Health Sciences, 23280 Elazig, Turkey; busraagenc.8@gmail.com (B.G.U.); drsozturk@gmail.com (S.Ö.); 2 Department of Biochemistry, Faculty of Medicine, Firat University, 23119 Elazig, Turkey; drseldatelo@hotmail.com

**Keywords:** psoriasis, oxidative stress, ADMA, MDA, glutathione

## Abstract

*Background and Objectives:* Psoriasis vulgaris (PV) is a chronic inflammatory disease associated with oxidative stress. It has been reported that oxidative stress caused by disruption of redox signaling can cause molecular damage, activate dendritic cells, lymphocytes, and keratinocytes, and lead to angiogenesis, inflammation, cell necrosis, and apoptosis by increasing the levels of lipid peroxidation products. In this study, serum levels of asymmetric dimethylarginine (ADMA), malondialdehyde (MDA), and reduced glutathione (GSH) were analyzed to gain insight into the oxidative balance in patients with PV. *Materials and Methods:* This prospective study included 59 PV patients and 40 healthy volunteers as the healthy control group. Age, gender, body mass index (BMI), waist circumference, routine hematologic parameters [fasting blood glucose, C-reactive protein (CRP), erythrocyte sedimentation rate (ESR), blood lipid levels, hemogram parameters], disease duration, and disease severity were recorded on data forms. The levels of ADMA, MDA, and GSH were analyzed using the high-performance liquid chromatography (HPLC) method. *Results:* When analyzed in terms of demographic characteristics, no statistically significant difference was observed between the patient and control groups. When examined in terms of biochemical variables, white blood cell (WBC) values were found to be significantly higher in the patient group (t: 2.825; *p* < 0.05). Although waist circumference, BMI, glucose, CRP, ESR, lipids, platelet count, and systolic and diastolic blood pressure were higher in the patient group, this difference was not statistically significant (*p* > 0.05). ADMA (t: 4.532; *p* < 0.05) and MDA (t: 9.598; *p* < 0.05) values were found to be higher and GSH (t: −4.717; *p* < 0.05) values were found to be lower in the patient group compared to the control group. When correlation analysis was performed between the parameters, a significant relationship was found only between GSH values and ADMA values (r: −0.256; *p* < 0.05). Accordingly, as the patients’ GSH values increased, ADMA values decreased. *Conclusions:* Increased WBC, ADMA, and MDA levels, and decreased GSH levels in PV patients reveal the critical role of oxidative stress and inflammation in the disease process. Evaluation of these biomarkers may contribute to the identification of new targets for the treatment of PV and the development of more effective management strategies.

## 1. Introduction

Psoriasis vulgaris (PV) is a chronic, inflammatory skin disease characterized by erythematous, psoriatic squamous plaques with clear borders [1].

It affects 1–3% of the population on average [2]. Although the pathogenesis of PV has not yet been fully elucidated, oxidative stress, genetic, inflammation, environmental, and immunologic factors are the most well-known [2]. Comorbidities such as hypertension, diabetes mellitus, dyslipidemia, metabolic syndrome, and cardiovascular disease are increased in PV [2]. These associations are thought to be mediated by shared inflammatory pathways and systemic immune dysregulation [3]. The chronic systemic inflammation characteristic of PV contributes to endothelial dysfunction, insulin resistance, and dyslipidemia, thereby linking it to these comorbid conditions [3].

Oxidative stress plays a role in the etiopathogenesis of many chronic diseases, including PV [4]. Oxidative stress plays a crucial role in the pathophysiology of PV by promoting keratinocyte hyperproliferation, inflammation, and immune dysregulation. Reactive oxygen species (ROS) activate NF-κB and other redox-sensitive transcription factors, which in turn enhance the expression of pro-inflammatory cytokines such as TNF-α, IL-17, and IL-23—key players in the PV inflammatory cascade [5].

It has been found that free oxygen radicals and oxidative stress play an important role in the pathophysiology of PV and are associated with disease severity [6]. Oxidative stress causes effects such as abnormal differentiation of keratinocytes, inflammatory cell infiltration, dysregulation of antioxidant activity, inhibition of the production of proinflammatory cytokines, and cell damage [6]. Oxidative stress markers have been found to be elevated in psoriasis and associated with the course and severity of the disease [7].

Nitric oxide (NO) is synthesized by nitric oxide synthase (NOS). Asymmetric dimethylarginine (ADMA) strongly inhibits NOS, decreasing NO production from L-arginine and lowering circulating NO levels. High NO levels decrease cell proliferation, while low NO levels increase keratinocyte proliferation [2,8]. In psoriatic lesions, the expression of inducible nitric oxide synthase (iNOS) is increased. This enzyme is stimulated by inflammatory cytokines such as IL-1β and TNF-α, leading to excessive NO production. Elevated NO levels contribute to the development of psoriatic lesions by promoting keratinocyte proliferation and inflammation [9,10].

ADMA is an endogenous molecule that can be detected in human blood and urine. High plasma ADMA concentrations have been shown to play a role in the pathophysiology of endothelial dysfunction, oxidative stress, inflammation, atherosclerosis, and impaired immunologic activity [8].

ADMA, which is thought to partially mediate the relationship between the inflammatory process and endothelial dysfunction in PV patients, has maintained its popularity in recent years both as an indicator of cardiovascular risk and as a potential regulator of the L-arginine-NO pathway [2]. Increased oxidative stress may elevate ADMA levels. High serum ADMA concentrations suggest that vascular oxidative stress increases in response to overall oxidative stress. However, the exact relationship between serum ADMA and oxidative stress remains unclear. Dimethylarginine dimethylaminohydrolase (DDAH), the enzyme responsible for ADMA degradation, shows reduced activity under oxidative stress, making it sensitive to redox imbalances. As a result, oxidative stress both increases ADMA production and impairs its degradation [11,12]. ADMA levels may be exacerbated by oxidative stress, which inhibits DDAH. Therefore, elevated ADMA may indirectly reflect existing oxidative stress [13].

The reaction of free oxygen radicals with lipids is generally known as “lipid peroxidation”. Malondialdehyde (MDA) is a widely accepted biomarker of oxidative stress, namely lipid peroxidation [14]. MDA is formed by the oxidation of polyunsaturated fatty acids in the cell membrane due to free radicals. This reflects oxidative damage to cellular membranes. MDA can disrupt various physiologic mechanisms of the human body by reacting with DNA and various proteins. Elevated MDA levels indicate increased oxidative damage, and MDA measurements are thus commonly used in oxidative stress assessments [15].

It has been reported that MDA protein elevations cause activation of Th17 lymphocytes, stimulate the release of various proinflammatory cytokines, and may trigger autoimmune reactions. In addition, increased MDA levels may lead to PV lesions in this way [16].

Reduced glutathione (GSH) is a tripeptide composed of glycine, glutamate, and cysteine. GSH is the major endogenous antioxidant commonly found in the body. Other antioxidants, such as selenium, vitamin E, and vitamin C, require GSH to perform their functions properly [17]. Glutathione has been reported to reduce the levels of free oxygen radicals and exert anti-inflammatory effects in hyperproliferative keratinocytes [18].

Disruption of glutathione metabolism is thought to contribute to the pathogenesis of PV by affecting various cells such as keratinocytes, macrophages, and dendritic cells, creating dysregulation in the oxidative–antioxidative system and imbalance in the inflammatory response [19]. GSH is the most abundant intracellular antioxidant, and its depletion is considered a marker of oxidative stress. GSH neutralizes reactive oxygen species and counters oxidative stress [20].

In this study, we aimed to investigate ADMA, MDA, and GSH levels in PV patients and evaluate the relationship between these biochemical parameters and clinical variables such as severity and duration of PV disease.

## 2. Methods

Approval for the study was obtained from the local ethics committee (2024/12-38). Participants who applied to the Dermatology Clinic at Fethi Sekin City Hospital between September 2024 and March 2025 were informed about the study, and both verbal and written consent were obtained. Patients aged between 18 and 65 years who were diagnosed with PV by a dermatologist based on clinical and/or histopathological criteria were included in the study. Eligibility criteria for the patient group were: absence of any other dermatological or systemic diseases, no pregnancy, no alcohol consumption, and no systemic drug use within the last three months. Patients receiving only topical treatments were eligible. Exclusion criteria included systemic treatment for PV (e.g., methotrexate, cyclosporine, biologics) and history of chronic systemic disease. Age, gender, body mass index (BMI), waist circumference, routine hematologic parameters [fasting blood glucose, C-reactive protein (CRP), erythrocyte sedimentation rate (ESR), blood lipid levels, hemogram parameters], disease duration, and disease severity were recorded on data forms. Disease severity was evaluated using the psoriasis area and severity index (PASI) measured by the same dermatologist [21]. Age- and sex-matched healthy volunteers without any dermatologic or systemic disease, pregnancy, alcohol use, or systemic drug use were recruited as the control group.

In order to determine the adequacy of the sample size in this study, a power analysis was conducted using the GPower program [22]. The power analysis performed with GPower yielded an “Actual Power” of 0.9537485. Accordingly, the required sample size was determined to be 24 participants for each group. An actual power value above 0.90 indicates that the sample size was sufficient.

In the morning, on an empty stomach, a 5 mL venous blood sample was collected from each participant in plain biochemistry tubes. The blood samples were immediately centrifuged, and the plasma obtained was stored at −20 °C. ADMA, MDA, and GSH levels in the collected serum samples were determined using the high-performance liquid chromatography (HPLC) method in accordance with the procedure specified in the manufacturer’s catalog.

ADMA levels were measured using a HPLC instrument using commercial kits (EUREKA srl-Laboratory division, Chiaravalle, Italy). ADMA was analyzed by HPLC following plasma purification and derivatization. Derivatization was performed using Reagent L, reconstituted in acetonitrile, as per the manufacturer’s protocol. After derivatization, 100 µL of solution was injected in HPLC system and analyzed by fluorescence detection. The chromatographic separation was carried out using a Phenyl Spherisorb reversed-phase column (250 × 4.6 mm, 5 µm). The mobile phase consisted of a proprietary buffer and methanol, delivered isocratically at a flow rate of 1.0 mL/min. ADMA was quantified using a HPLC system with a spectrofluorometer detector set at 483 nm (emission) and 420 nm (excitation). Results were expressed in micromoles per liter.

The level of MDA, as an index of lipid peroxidation, was analyzed using an MDA kit (Immuchrom GmbH, Hessen, Germany) with HPLC. Initially, protein-bound MDA was hydrolyzed and converted into a fluorescent product by incubation at 95 °C for 60 min. For MDA measurement, derivatization was performed using 2,4-dinitrophenylhydrazine. The product was then cooled (2–8 °C), centrifuged, mixed with a reaction solution, and injected into the HPLC system. MDA-generated fluorescence was measured using an isocratic HPLC system with a spectrofluorometric detector at 515 nm (excitation) and 553 nm (emission). Separation was performed on a Prontosil Eurobond reversed-phase column (125 × 4 mm, 5 µm). The mobile phase, provided by the manufacturer, was used in isocratic mode with a flow rate of 0.8–1.0 mL/min. Results were expressed in micromoles per liter (µmol/L). The use of HPLC for MDA determination has been previously validated and shown to be reliable in biological matrices, including brain tissue, as demonstrated in a recent method validation study [23].

GSH levels were measured using a GSH kit (Immuchrom GmbH, Hessen, Germany) with HPLC (Shimadzu RF-10AxL). During the derivatization reaction, GSH was converted into a fluorescent probe. A fluorogenic reagent, ammonium 7-fluorobenzo-2-oxa-1,3-diazole-4-sulfonate (SBD-F), was used as the derivatizing reagent for the determination of GSH. The precipitation step removed high molecular weight substances. After centrifugation, the fluorescent probe was cooled (2–8 °C) and a 20 μL sample was injected into the HPLC system. The mobile phase consisted of a methanol–water mixture, delivered at a flow rate of 0.75 mL/min. Measurements were carried out on the HPLC system with a fluorescence detector at 385 nm (excitation) and 515 nm (emission). Results were expressed in micromoles per liter.

### Statistical Analysis

The Statistical Package for Social Science for Windows (SPSS) 26.0 package program was used to evaluate the data obtained in the study. Frequency and percentage distributions were analyzed to determine the gender distribution, mean and standard deviation values for age, height, weight, BMI, and waist circumference values. Mean and standard deviation values were analyzed to determine the biochemical parameters and other variables. Independent Samples *t*-test analysis was applied to examine whether ADMA, MDA, and GSH values, which constitute the main purpose of the study, showed a statistically significant difference between the patient and control group. Comparisons of ADMA, MDA, and GSH levels between genders and across different age groups were performed using the Independent Samples *t*-test. In addition, Spearman’s correlation analysis was applied to examine statistically significant relationships between ADMA, MDA, and GSH values in the patients. The results were considered significant at the 95% confidence level (*p* < 0.05).

## 3. Results

A total of 59 patients diagnosed with psoriasis vulgaris and 40 healthy individuals who met the inclusion criteria were included in the study. When analyzed in terms of demographic characteristics, no statistically significant difference was observed between the patient and control groups (Table 1). When analyzed in terms of biochemical variables, a significant difference was found only in terms of WBC values (t:2.825; *p* < 0.05). Accordingly, the WBC values of the patient group (7640.67 ± 1852.40) were higher than the WBC values of the control group (6562.60 ± 1879.98). Although waist circumference, BMI, glucose, CRP, ESR, lipids, platelet count, and systolic and diastolic blood pressure were higher in the patient group, this difference was not statistically significant (*p* > 0.05) (Table 2).

Statistically significant differences were observed between the patient and control groups in ADMA, MDA, and GSH levels (Table 3).

When correlation analysis was performed between GSH, ADMA, and MDA levels and PASI of the patients, a significant relationship was found only between GSH values and ADMA values (r: −0.256, *p* < 0.05). Accordingly, ADMA values decreased as the GSH values of the patients increased. No correlation was found between the other parameters.

When ADMA, MDA, and glutathione levels were analyzed according to age groups within the patient and control groups, no statistically significant differences were observed across age groups in terms of ADMA, MDA, or glutathione levels, in either the patient or control groups (*p* > 0.05). When the ADMA, MDA and GSH levels were compared by gender within the patient and control groups, in the patient group, a statistically significant gender-based difference was observed in MDA (t: 2.604; *p* < 0.05) and GSH levels (t: 2.736, *p* < 0.05). No significant gender-based differences were found in ADMA levels in either the patient or control groups. Regarding MDA levels, no significant gender difference was found in the control group. However, in the patient group, female patients (2.53 ± 0.59) had significantly higher MDA levels compared to male patients (2.07 ± 0.77). Similarly, no significant gender difference was found in GSH levels in the control group, but among patients, females (8.67 ± 3.73) had significantly higher GSH levels compared to males (6.44 ± 1.86). No significant difference was found between PASI and gender (*p* > 0.05).

## 4. Discussion

Oxidative stress occurs when the balance between ROS and antioxidant defense mechanisms is disrupted and can lead to cellular damage. In recent years, the importance of oxidative stress in the development of PV has been emphasized. It has been reported that oxidative stress caused by disruption of redox signaling can cause molecular damage, activate dendritic cells, lymphocytes, and keratinocytes, and lead to angiogenesis, inflammation, cell necrosis, and apoptosis by increasing the levels of lipid peroxidation products [24].

It is known that PV stimulates proinflammatory responses and chronic inflammation through activation of monocytes, macrophages, neutrophils, and endothelial cells, and leads to cytokine and ROS production as a result of activation of the immune system [25].

Zhou et al. conducted a recent retrospective study of 361,322 psoriasis patients from the UK Biobank [UKB] and 3971 psoriasis patients from China to examine the association between circulating white blood cells and psoriasis. As a result of the study, they found a definite relationship between WBC elevation and WBC subgroups and psoriasis. They reported that this result may be helpful to some extent in dermato-epidemiology and clinical practice [26]. In our study, similar to this study and many studies in the literature, WBC levels were found to be significantly higher in the patient group [26,27,28]. Although waist circumference, BMI, glucose, CRP, ESR, lipids, platelet count, and systolic and diastolic blood pressure were higher in the patient group as expected, this difference was not statistically significant, similar to some studies [29,30].

In a study conducted on psoriasis patients, it was found that total ROS content and lipoperoxidation levels in three cell groups, including granulocytes, monocytes, and lymphocytes, were significantly higher than controls [31]. Many studies reported that total oxidant capacity and oxidative stress index were higher, and total antioxidant capacity levels were lower in psoriasis patients compared to the control group [31,32,33,34].

Various studies have also found an increased risk of cardiovascular disease in psoriasis [35,36]. In this study, the levels of ADMA [37], which is found to cause vascular endothelial dysfunction and increase inflammatory markers and ROS production, MDA [14], which is accepted as a marker of lipid peroxidation, and GSH [18], an important component of the antioxidant system, were investigated in psoriasis patients.

ADMA is an endogenous inhibitor of NO synthase, and increased levels may impair NO production, leading to endothelial dysfunction and an increased risk of cardiovascular disease [36]. One study showed that serum ADMA levels increased in psoriasis patients, and this increase was strongly associated with PASI scores. It has been suggested that the arginine–NO pathway may play an important role in the pathogenesis of psoriasis, and ADMA may be associated with disease severity [2].

In the study by Pohla et al. [38], lesional and non-lesional skin biopsies from plaque psoriasis patients were compared with healthy controls and ADMA levels in lesional skin were found to be significantly higher.

In a study including 29 psoriasis patients, ADMA levels were found to be positively correlated with disease severity [39]. In another study conducted in Türkiye, ADMA levels were evaluated in 40 male PV patients aged 20–41 years and 40 healthy male volunteers aged 20–32 years. Although the ADMA level was higher in the patient group, it was not statistically significant. Again, no significant relationship was found between ADMA levels and disease severity [40]. The reason for the lack of a significant difference between PV and ADMA levels in this study may be the inclusion of younger age and only male gender compared to our study and similar studies.

In acne vulgaris, an inflammatory disease associated with oxidative stress, ADMA levels were found to be higher in the patient group compared to healthy controls and positively correlated with disease severity [41]. In our study, similar to many studies above, ADMA levels were significantly higher in patients with PV. Again, in our study, ADMA levels increased with increasing disease severity, similar to some studies, but there was no statistically significant correlation.

Oxidative stress causes peroxidation of polyunsaturated fatty acids in cell membranes. In this process, ROS reacts with membrane lipids to form reactive aldehydes. These reactive aldehydes, such as MDA formed during lipid peroxidation, covalently bind with proteins to form adducts. These modifications negatively affect cellular processes by disrupting the structure and function of proteins. Enzyme inhibition may lead to inactivation of enzymes involved in cellular antioxidant defense. They may disrupt cell signaling, leading to irregularities in keratinocyte proliferation and differentiation. Modified proteins can activate the immune system and trigger an inflammatory response. This initiates processes such as T-cell activation and increased release of inflammatory cytokines [e.g., TNF-α, IL-17, IL-23], which play a critical role in the pathogenesis of psoriasis. Protein adducts can accumulate in the epidermis and disrupt keratinocyte function, leading to hyperproliferation, impairment of skin barrier function, and the formation of clinical psoriatic plaques. In addition, these lipid peroxidation products and modified proteins are not limited to local effects but may also enter the circulation and contribute to systemic inflammation and comorbidities of psoriasis [e.g., cardiovascular diseases] [16]. Oxidative stress plays a role as both a cause and a consequence in psoriasis. Environmental factors and genetic predisposition may contribute to the onset of the disease by increasing oxidative stress. At the same time, psoriatic inflammation further amplifies oxidative stress by enhancing the production of ROS [42].

A study by Wadhwa et al. found that serum MDA levels in patients with psoriasis were significantly higher than in healthy individuals, indicating increased lipid peroxidation [43]. In a meta-analysis by Cannavò et al., which included 28 studies examining the role of oxidative stress in psoriasis, several molecules were shown to be associated with the disease, but a small group of them, including MDA, were considered suitable as disease biomarkers. In addition, only MDA was considered to be the most suitable candidate for clinical screening of psoriasis as it is closely linked to PASI [44]. In our study, MDA levels were found to be significantly higher in the patient group. However, this increase was not statistically significant, although it increased in direct proportion to disease severity. This may be related to the fact that we could not include very severe forms of the disease in our study because of systemic drug use.

GSH has been one of the focal points of scientific interest in recent years. In the human body and other living organisms, it plays an important role in all important physiological processes by participating in cellular redox reactions and providing antioxidant defenses. GSH also plays important roles in the detoxification of xenobiotics, protection of protein thiols from cross-linking and oxidation, regulation of the cell cycle, reduction of oxidative stress, enhancement of metabolic detoxification, and regulation of immune system function. It is also said that keeping GSH levels in balance can treat some age-related disorders. Because of all these properties, GSH has been reported to be a universal biomarker [45].

Low GSH levels in psoriasis patients indicate that antioxidant defense capacity is insufficient. A decrease in GSH levels may lead to increased oxidative stress and the release of inflammatory mediators [17]. Glutathione S-transferase and glutathione peroxidase, which are enzymes that conjugate GSH with substrates and neutralize the harmful effects of peroxides by using GSH, were investigated in PV patients. It has been reported that low levels or activities of these enzymes may contribute to the pathogenesis of PV due to inadequate antioxidant protection [18,46].

The significantly lower GSH levels of PV patients in our study indicate that antioxidant capacity is decreased and the oxidative stress burden is increased in these patients. GSH deficiency may contribute to increased inflammatory processes and PV progression.

In our correlation analysis, a significant negative correlation was found between GSH levels and ADMA in PV patients. ADMA levels decreased as GSH levels increased. This relationship indicates that increased antioxidant capacity may prevent endothelial dysfunction. The protective effects of GSH on inflammation and oxidative stress may be an important mechanism for reducing cardiovascular risks in PV patients by lowering ADMA levels.

## 5. Conclusions

In conclusion, increased WBC, ADMA, and MDA levels and decreased GSH levels in PV patients reveal the critical role of oxidative stress and inflammation in the disease process. Evaluation of these biomarkers may contribute to the identification of new targets for the treatment of PV and the development of more effective management strategies.

These findings suggest that PV is not a disease limited to the skin and that systemic inflammation and oxidative stress play a central role in the pathogenesis of the disease. Strategies to reduce oxidative stress in the treatment of PV may have positive effects on patients’ overall health. For example, antioxidant therapies that increase GSH levels or pharmacologic agents that lower ADMA and MDA levels should be evaluated in future clinical trials.

### Limitations

Our results are consistent with previous findings in the literature. However, several limitations should be acknowledged. First, the relatively small sample size may limit the generalizability of the results. Second, the absence of long-term, prospective follow-up data restricts conclusions regarding the stability and progression of oxidative stress markers over time. In addition, certain potential confounding factors such as lifestyle habits (e.g., smoking, diet, and exercise) and comorbidities were not thoroughly controlled for, which may have influenced the outcomes. Furthermore, although GSH was measured, oxidized glutathione (GSSG) could not be analyzed due to technical limitations, as commonly reported in similar studies. Future research involving larger populations, prospective designs, and comprehensive assessment of oxidative stress parameters is needed to confirm and expand upon these results.

## Figures and Tables

**Table 1:** Comparison of patient and control group baseline data.

Variables	Patient (n = 59)	Control (n = 40)	t	*p*
Gender (Female/Male)	34/25	28/12	1.559 **	0.150
Age (years)	41.38 ± 13.06	36.60 ± 11.50	1.877	0.064
Height (cm)	166.54 ± 8.28	167.92 ± 9.71	−0.760	0.449
Weight (kg)	75.64 ± 14.78	72.65 ± 13.38	1.027	0.307
BMI (kg/m^2^)	27.04 ± 4.83	25.71 ± 3.84	1.449	0.150
Waist Circumference (cm)	96.55 ± 16.65	83.67 ± 12.17	1.189	0.114
Systolic BP (mmHg)	126.23 ± 14.58	116.23 ± 12.76	1.489	0.238
Diastolic BP (mmHg)	77.44 ± 10.26	75.35 ± 8.33	1.057	0.273

Data are presented as mean ± standard deviation (SD) unless otherwise indicated. t: Independent Samples *t*-test; ** Χ^2^: Chi-square test. *p* < 0.05 was considered statistically significant. BMI: body mass index; BP: blood pressure.

**Table 2:** Comparison of biochemical parameters between patient and control groups.

Variables	Patient (n = 59)	Control (n = 40)	t	*p*
Glucose (mg/dL)	95.71 ± 33.50	89.77 ± 14.94	1.051	0.296
Total Cholesterol (mg/dL)	176.25 ± 38.11	173.20 ± 36.57	0.398	0.692
HDL (mg/dL)	52.52 ± 18.13	48.62 ± 11.39	1.207	0.230
LDL (mg/dL)	105.13 ± 46.89	100.75 ± 35.15	0.503	0.616
Triglycerides (mg/dL)	138.37 ± 98.88	129.00 ± 65.87	0.525	0.601
CRP (mg/L)	4.86 ± 1.85	3.78 ± 1.96	0.726	0.470
ESR (mm/h)	7.20 ± 5.45	6.72 ± 5.33	0.432	0.667
Hemoglobin (g/dL)	14.19 ± 1.55	14.20 ± 1.49	−0.149	0.882
WBC (cells/mm^3^)	7640.67 ± 1852.40	6562.60 ± 1879.98	2.825	0.006 *
PLT (cells/mm^3^)	281.15 ± 58.47	262.65 ± 55.20	1.580	0.117

Data are presented as mean ± standard deviation (SD) unless otherwise indicated. t: Independent Samples *t*-test, * *p* < 0.05; CRP, C-reactive protein; ESR, erythrocyte sedimentation rate; WBC, white blood cell; HDL, high-density lipoprotein; LDL, low-density lipoprotein; PLT, platelet.

**Table 3:** Comparison of oxidative stress parameters.

Variables	Patient (n = 59)	Control (n = 40)	t	*p*
ADMA (µmol/L)	1.15 ± 0.43	0.76 ± 0.39	4.532	<0.001 *
MDA (nmol/mL)	2.34 ± 0.70	1.11 ± 0.45	9.598	<0.001 *
GSH (µmol/L)	7.73 ± 3.25	16.43 ± 5.63	−4.717	<0.001 *

Data are presented as mean ± standard deviation (SD) unless otherwise indicated. t: Independent Samples *t*-test, * *p* < 0.05; ADMA, asymmetric dimethylarginine; MDA, malondialdehyde; GSH, glutathione.

## Data Availability

The data presented in this study are available on request from the corresponding author.
